# Study of Folliculometry After Spontaneous and Clomiphene Citrate-Induced Ovulation in Infertile Women

**DOI:** 10.7759/cureus.42234

**Published:** 2023-07-21

**Authors:** Smita Singh, Uday Prakash

**Affiliations:** 1 Obstetrics and Gynecology, All India Institute of Medical Sciences, Patna, Patna, IND; 2 Orthopedics and Traumatology, Darbhanga Medical College, Darbhanga, IND

**Keywords:** anovulation, clomiphene citrate, ovulation induction, transvaginal ultrasound, infertility, oligo-ovulation

## Abstract

Background

Ovulatory disruption is the primary reversible cause of infertility, which affects 12-24% of couples. The FDA's first-line recommendation for ovulation induction in such cases is clomiphene citrate. Serial ultrasonography can be used to evaluate follicular development.

Methodology

The current study is a two-year prospective cohort study conducted at a tertiary care centre. One hundred patients with either primary or secondary infertility and no pelvic pathology were involved in the study and split into two groups: Group I included ovulatory women whose infertility was caused by a factor other than ovulatory disorder, and Group II comprised anovulatory women. Folliculometry was performed using transvaginal sonography; Group I had a spontaneous cycle, whereas Group II received clomiphene citrate (CC) to induce ovulation. The ovulation rate, pregnancy rate, multiple pregnancy rate, and rate of ovarian hyperstimulation (OHSS) were all studied.

Results

Seventy-two percent of the patients had primary infertility, and most appeared after 3-6 years of infertility. 62% of the patients were between the ages of 21 and 30 years. 50% of cases had ovulatory dysfunction, and polycystic ovarian disease (PCOD) was the most frequent cause of anovulation (24%). The leading follicular diameter was substantially bigger (22-26 mm) in the CC-triggered cycle compared to the spontaneous cycle (16-21 mm). In both spontaneous and induced cycles, the endometrial thickness displayed a linear development pattern during the pre-ovulatory phase and plateaued during the luteal phase. With CC, there was a 68% ovulation rate, a 32% pregnancy rate, a 12.5% multiple pregnancy rate, and a 2% incidence of OHSS.

Conclusion

Clomiphene citrate increases the rate of ovulation and pregnancy in females having ovulatory disorders.

## Introduction

According to recent population-based research, infertility affects between 12% and 24% of couples [1]. Given that this prevalence is higher in older women (>35 years old), infertility evaluation should start in women between the ages of 35 and 40 after six months, and immediately in those over 40 [2]. Infertility affects 10-15% of the population, with female factors accounting for 40-45%, male factors accounting for 25-40%, both male and female factors accounting for 10%, and the remaining 10% having no known cause [3-5]. Between 30% and 40% of female infertility is caused by ovulatory dysfunction, polycystic ovary syndrome (PCOD) being the most common [6-8].

For many years, only clinical indicators were used to monitor ovulation. Ultrasonographic imaging was first developed by Donald (1950) [9], but its application was sparse until Kratochwit et al. (1972) first documented visualising and tracking follicular growth throughout the ovarian cycle [10]. Hackeloer (1979) used ultrasonography to demonstrate the sequential processes that occur in an ovary throughout the normal menstrual cycle and discovered a linear link between the follicular size and plasma estrogen levels for predicting ovulation [11, 12]. This study aimed to study the follicular dynamics after spontaneous and induced ovulation by a noninvasive method in infertile women of the reproductive age group.

## Materials and methods

Study design and setting

This is a prospective cohort study that was undertaken at a tertiary care centre for two years.

Participants

The selection of patients for the study was done from patients attending the Gynecology Patient's Department for Infertility, either primary or secondary. All females between the ages of 21 and 40 years who had been unable to conceive for more than a year while having unprotected intercourse and were willing to participate in the trial were screened for various causes of infertility. The patients were examined clinically and relevant histories were recorded. Everyone willing to take part was enrolled, and those who were not ready or had any pelvic pathology were excluded from the study.

Following the screening, 100 infertile patients were chosen for the study and split into two groups: Group I consisted of 50 infertile women with ovulatory cycles whose infertility was due to something other than ovulatory dysfunction. Group II consisted of 50 infertile women with anovulatory cycles. Detailed studies of follicular dynamics were conducted in both groups. The success and failure rates of therapy, as well as the dangers of multiple pregnancies and ectopic pregnancies, were explained to the couples.

In Group II, clomiphene citrate (CC) was given to stimulate ovulation. Women with regular cycles were asked when their menstruation began, and women with amenorrhoea were given progesterone medication for withdrawal bleeding. In all cases, ovulation treatment began on D2/D3 of the cycle. 50 mg of clomiphene citrate was given daily from D2 to D6 of the menstrual cycle to trigger ovulation. If ovulation did not occur, the dose was increased to 100 mg per day the following cycle, and then to a maximum of 150 mg per day the cycle after that. Transvaginal ultrasonography was used to assess follicles starting on D10 of the cycle and continuing on alternate days till rupture. The number of follicles, serial rise in follicle diameter, rate of growth, and thickness of the endometrium were all noted. Ovulation is confirmed by transvaginal sonography (TVS) by the following signs: (1) complete disappearance of the follicles, (2) follicular wall irregularity, (3) appearance of several echoes in a previously echo-free follicle, (4) presence of fluid in the pouch of Douglas (POD), and (5) endometrium with hyperechogenic secretory tissue.

After the human chorionic gonadotropin (HCG) trigger, timed intercourse was advised. The occurrence of ovarian hyperstimulation (OHSS) was also investigated, and based on the following findings, OHSS was identified.

Mild OHSS was subcategorised as: Ovarian enlargement with - Grade 1 - abdominal distension and discomfort; and Grade 2 - features of grade 1 + nausea, vomiting and/or diarrhoea, and ovaries are enlarged (<5 cm).

Moderate OHSS was subcategorised as: Grade 3 - features of mild OHSS + mild abdominal distention + ultrasonographic evidence of ascites; and Grade 4 - gastrointestinal upset and ovarian enlargement (5-12 cm).

Severe OHSS was subcategorised as: Grade 5 - features of moderate OHSS + tense ascites and/or hydrothorax and breathing difficulties; and Grade 6 - all the features of Grade 5 + changes in blood volume, increased blood viscosity due to hemoconcentration, coagulation abnormalities, respiratory failure, and diminished renal perfusion and function.

The patients were followed up on and asked to report if they missed their period, and pregnancy was verified using a urine pregnancy test and transvaginal sonography. The pregnancy rate and multiple pregnancy rates were analysed. Data analysis was done using Microsoft Excel (Microsoft Corporation, Redmond, USA).

## Results

Seventy-two percent of the patients experienced primary infertility. 62% of the patients were between the ages of 21 and 30 years, while 38% were between the ages of 31 and 40 years. The greatest percentage of instances (78%) were documented after a 3 to 6-year infertility interval. 40% of them had regular menstrual cycles, with oligomenorrhea (36%) being the most frequent menstrual irregularity (Table 1).

**Table 1:** Patient characteristics

	No. of Patients (n=100)	% of patients
1. Type of infertility		
Primary	72	72%
Secondary	28	72%
2. Age in years		
21-25	22	22%
26-30	40	40%
31-35	31	31%
36-40	7	7%
3. Duration of infertility in years		
1-2	4	4%
3-4	38	38%
5-6	40	40%
7-8	16	16%
9-10	2	2%
4. Menstrual pattern		
Amenorrhea	10	10%
Oligomenorrhea	36	36%
Menorrhagia	14	14%
Normal menstrual cycle	40	40%

Ovulatory dysfunction, which accounted for 50% of cases, was the most common cause of infertility overall (Table 2).

**Table 2:** Etiological distribution

Etiologic factor	No. of Patients	% of patients
Ovulatory factor	50	50%
Tubal factor	20	20%
Uterine factor	4	4%
Cervical factor	4	4%
Male factor	10	10%
Unexplained infertility	12	12%

**Figure 1:**
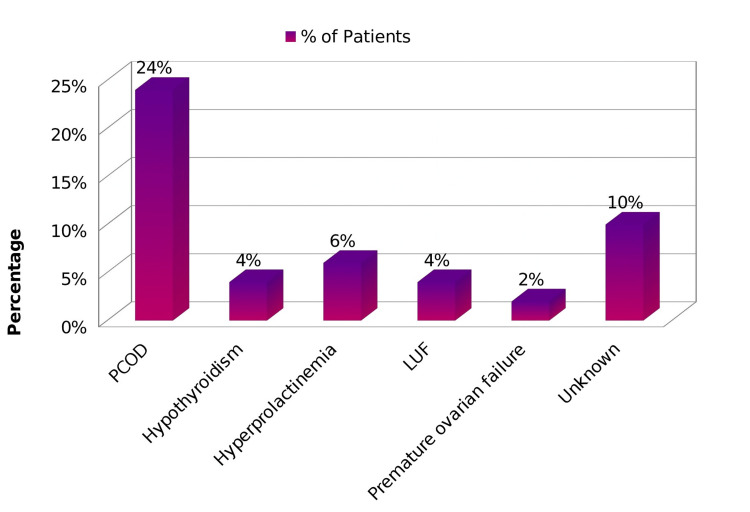
Classification of the causes of anovulation PCOD: Polycystic Ovarian Disease

The most common cause of anovulation was PCOD (24%) (Figure 1).

In 88% of cases (Table 3), ovulation in spontaneous cycles was determined by the disappearance of the follicle and the emergence of fluid in the POD. In contrast, in induced cycles, ovulation was determined by the disappearance of the follicle (36%) and the presence of fluid in the POD (52%) (Table 4).

**Table 3:** Study of follicular dynamics by USG in spontaneous ovulatory cycles USG: ultrasonography; POD: pouch of Douglas

Ultrasound signs of ovulation	No. of Patients	% of patients
1. Appearance of multiple echoes in a previously echo-free follicle	4	8%
2. Change in shape	2	4%
3. Disappearance of follicle	20	40%
4. Appearance of fluid in POD	24	48%
	N = 50	

**Table 4:** Study of follicular dynamics by USG after inducing ovulation by clomiphene citrate. USG: ultrasonography; POD: pouch of Douglas

Ultrasound signs of ovulation	No. of Patients	% of patients
1. Appearance of multiple echoes in a previously echo-free follicle	4	8%
2. Change in shape	2	4%
3. Disappearance of follicle	18	36%
4. Appearance of fluid in POD	26	52%
	N = 50	

The leading mean follicular diameter was substantially larger in stimulated cycles (22-24 mm) compared to spontaneous cycles (16-21 mm) (Table 5).

**Table 5:** Comparison of leading follicular diameter

Day before ovulation	Mean Follicular Diameter (in mm)	
	Spontaneous	Clomiphene Induced
Day – 3	16	22
Day – 2	19	24
Day – 1	21	26
	Spontaneous	Clomiphene
Average No. of ovulating follicles having diameter > 16 mm	1	2.5

**Figure 2:**
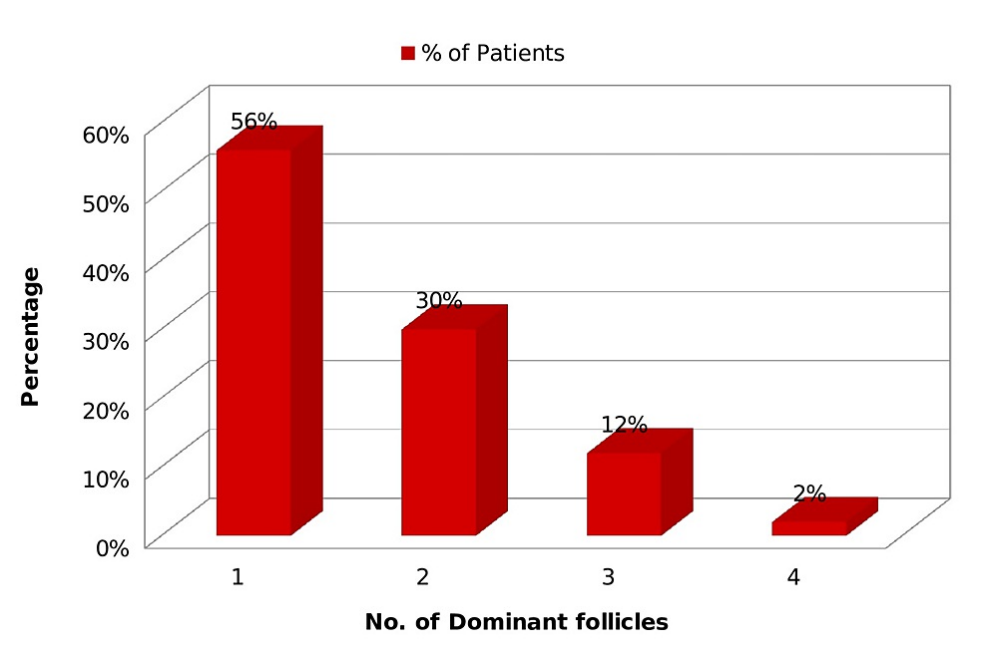
Number of dominant follicles in clomiphene citrate-induced cycles

During stimulated cycles, the ultrasonography (USG) observed multi-follicular development. One follicle is present in 56% of cases, followed by two follicles in 30%, three follicles in 12%, and four follicles in 2% of cases (Figure 2).

**Figure 3:**
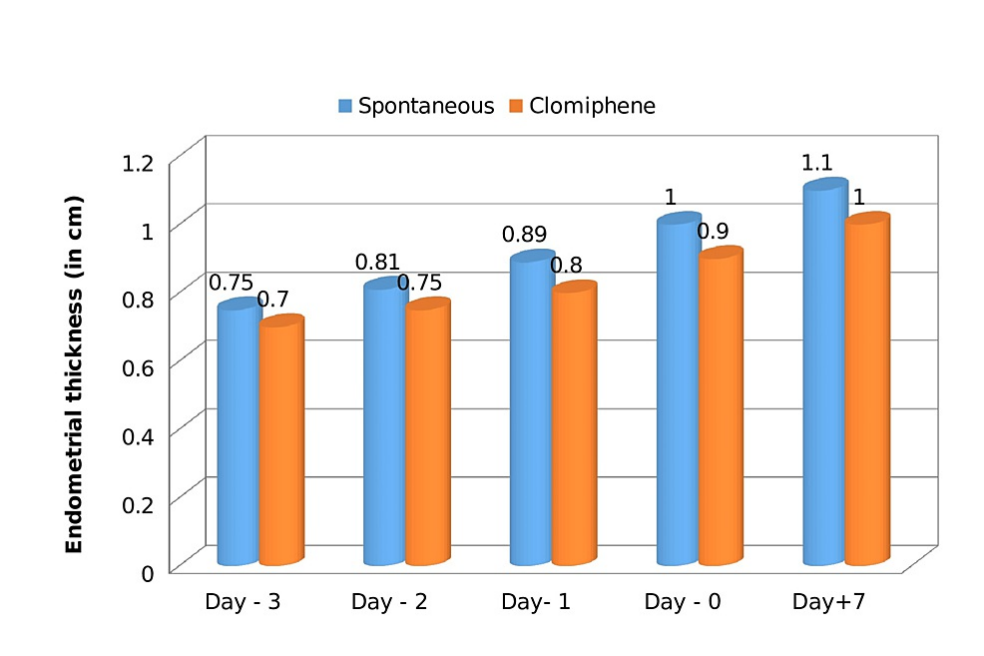
Comparison of endometrial thickness in cm (spontaneous vs. induced)

Endometrial thickness increased linearly during the pre-ovulatory phase of both spontaneous and induced cycles before plateauing during the luteal phase (Figure 3).

The ovulation rate was 68% (34), the pregnancy rate was 32% (16), and the multiple pregnancy rate was 12.5% (2) in the CC-induced group. Two individuals (4%), who had mild OHSS, were noted.

## Discussion

The most popular way of treating infertility is ovulation induction, which involves stimulating the ovaries to create many follicles. Clomiphene citrate is a selective oestrogen receptor modulator (SERM) that binds to oestrogen receptors in the hypothalamus, ovary, endometrial, and cervix, producing oestrogenic and anti-oestrogenic effects. It functions as a partial oestrogen agonist in the hypothalamus, inhibiting oestrogenic negative feedback and increasing gonadotropins [13]. It is a nonsteroidal chemical that indirectly stimulates ovulation, was synthesised in 1956, made available for clinical trials in 1960, and was licensed for use in clinical settings in the United States in 1967, leading to a revolution in the medical therapy of infertility [14]. It is particularly helpful for treating individuals with anovulatory disorders and polycystic ovary syndrome (PCOS) [15]. It has been used for decades in ovulation induction and assisted reproduction for up to 6 cycles [16]. It has been approved by FDA to treat anovulatory or oligo-ovulatory infertility in women who want to conceive. According to research, using clomiphene to induce pregnancy resulted in a 6-month live-birth rate of 20% to 40% [17] [14].

Studies show that about 30-40% of cases of female infertility are caused by ovulation disorders which are in line with our study [18, 19]. TVS is a rapid, simple, and noninvasive way of directly observing ovarian follicle development that has no negative impact on the oocyte or reproductive system. Serial ultrasound can be used to monitor follicular growth, detect follicular maturity at the time of HCG administration, and execute timed intercourse [20].

Debnath et al. found that the mean pre-ovulatory diameter in spontaneous cycles was 20 mm (range 15 to 29 mm) and 24 mm (range 16 to 32 mm) in CC cycles. In CC-stimulated cycles vs. spontaneous cycles, the average rate of follicular growth was 2.1 mm/day and 1.9 mm/day, respectively. In 52% of stimulation cycles, there was a multi-follicular response. Ovarian hyperstimulation syndrome was detected sonographically in 1.84% of cycles. All these findings are consistent with the findings of our study wherein the leading mean follicular diameter was significantly higher (22-24 mm) in stimulated cycles in contrast to spontaneous cycles (16-21 mm), the follicular growth rate was 2.1 mm/day and 2.2 mm/day, 44% had multi follicular development in the induced cycle and rate of OHSS was 4%, in spontaneous and induced cycles, respectively [18]. These results are in line with those of our study, which showed that the leading mean follicular diameter was considerably larger (22-24 mm) in stimulated cycles than in spontaneous cycles (16-21 mm). Follicular growth rates were 2.1 and 2.2 mm per day in spontaneous and induced cycles, respectively, while the rates of multi-follicular development and OHSS were 44% and 4%, respectively. The average growth rate of endometrium was 0.083 cm/day in the spontaneous cycle and 0.075 cm/day in an induced cycle, which is consistent with previous studies [12].

## Conclusions

An essential part of the aetiology of infertility is ovulatory dysfunction and anovulation. As a quick and simple way to examine ovarian follicle development, follicular dynamics investigation by serial ultrasonography is valuable for monitoring patients undergoing ovulation induction. Additionally, it is non-invasive, takes little time, and has no known negative effects on the oocyte or reproductive system. The proper prediction of ovulation timing is crucial for infertility therapies such as intrauterine insemination, artificial or therapeutic insemination utilising donor sperm, and the timing of intercourse. Clomiphene is the current first-line infertility treatment in women with ovulatory aetiology.
